# Multiplexed Targeted Spatial Mass Spectrometry Imaging Assays to monitor lipids and NAD^+^ metabolites in CD38 knockout mice exhibiting improved metabolism

**DOI:** 10.21203/rs.3.rs-6743284/v1

**Published:** 2025-06-16

**Authors:** Birgit Schilling, Charles Schurman, Joanna Bons, Prasanna Kumaar, Jingji Fang, Andrea Roberts, Genesis Hormazabal, Rebeccah Riley, Nannan Tao, Eric Verdin

**Affiliations:** Buck Institute for Research on Aging; University of California, San Francisco; Buck Institute for Research on Aging; Buck Institute for Research on Aging; Buck Institute for Research on Aging; Buck Institute for Research on Aging; Buck Institute for Research on Aging; Buck Institute Research on Aging; Bruker; Buck Institute for Research on Aging

## Abstract

Mass spectrometry imaging (MSI) is a rapidly advancing technology that provides mapping of the spatial molecular landscape of tissues for a variety of analytes. Matrix-assisted laser desorption/ionization (MALDI)-MSI is commonly employed, however, confident *in situ* identification and accurate quantification of analytes remain challenging. We present a novel imaging methodology combining trapped ion mobility spectrometry (TIMS)-based parallel accumulation-serial fragmentation (PASEF) with MALDI ionization for targeted imaging parallel reaction monitoring (iprm-PASEF). We investigated the spatial distribution of lipids and metabolites in liver tissues from wild-type and CD38 knockout mice (CD38^−/−^). CD38, an enzyme involved in nicotinamide adenine dinucleotide (NAD+) metabolism, significantly influences liver metabolic function and contributes to age-related NAD+ decline. Although CD38 deletion previously was linked to improved metabolic phenotypes, the underlying spatial metabolic mechanisms are poorly understood. The spatial iprm-PASEF workflow enabled confident identification and differentiation of lipid isomers at the MS2 fragment ion level and revealed increased NAD^+^ and decreased adenosine diphosphate ribose (ADPR), a by-product of NAD^+^ hydrolysis, in CD38^−/−^ livers. This approach provided confident, specific, and robust MS2-based identification and quantification of fragment ions in spatial MSI experiments. Additionally, the innovative iprm-PASEF opens unprecedented opportunities for spatial metabolomics and lipidomics, offering spatially resolved insights into molecular mechanisms.

## Introduction

Mass spectrometry imaging (MSI) is a rapidly advancing application of mass spectrometry (MS) allowing for the unbiased spatial assessment of a variety of analytes and biological samples. MSI promises the capability to image thousands of molecules, such as metabolites, lipids, peptides, proteins, and glycans in tissues, while preserving their original spatial tissue localization.^1^ This allows for the combination of the power, sensitivity, and specificity of mass spectrometry with spatial molecular details that can reveal novel biological regulation. Access to spatially resolved molecular features (proteins, glycans, lipids and metabolites) is instrumental to understand high tissue heterogeneity with localized hot spots of tissue damage, specifically during aging, (age-related) diseases and clinical applications. Using global proteomics/glycomics/lipidomics or metabolomics these spatial features are very challenging to capture, and indeed, often remain undetected or non-significant in ‘bulk-style’ analyses. On the other hand, sophisticated applications of spatial MS Imaging exist with multiple sources and ionization strategies, such as desorption electrospray ionization (DESI), laser ablation electrospray ionization (LAESI), liquid extraction surface analysis (LESA), liquid-micro junction-surface sampling probe (LMJ-SSP), infrared matrix-assisted laser desorption electrospray ionization (IR-MALDESI), and nanoscale secondary ion mass spectrometry (NanoSIMS).^2,3^ Another popular approach for MSI is matrix-assisted laser desorption/ionization (MALDI)-MSI which utilizes a deposited matrix-enhanced analyte ionization in place of laser ablation. MALDI-MSI allows for the discrete collection of pixel-specific mass spectra that are then assembled to generate a visual representation of the molecular species throughout the imaged region.

Several of the existing imaging techniques are limited to MS1 level analysis and do not utilize precursor ion fragmentation to increase identification confidence or improved quantification at the fragment ion (MS2) level. Or, in other cases MS/MS spatial imaging is often limited to selection of one analyte for MS/MS per acquisition covering a spatial region of interest (ROI). Powerful applications of MSI, such as DESI mass spectrometry, use a liquid-based probe to collect analytes in place and then analyze them by liquid chromatography-tandem mass spectrometry (LC-MS/MS) in a spatially resolved manner.^4^ However, DESI-MS may present challenges with humidity and extraction efficiencies, as well as present resolution challenges that stem from the continuous solvent flow and the size of the liquid bridge formed between the tissue sample and the DESI probe.^5^ Herein, we detail a novel workflow for MALDI-MSI that employs targeted imaging parallel reaction monitoring (iprm) in combination with PASEF that extends MALDI-MSI analysis from the MS1 level to the tandem mass spectrometry (MS2) dimension for *in situ* analysis of lipids and metabolites (or other analytes) in biological samples. Importantly, multiple MS/MS spectra can be acquired using iprm-PASEF in a single imaging tissue MSI workflow enabling multiplexed and targeted MS2-based spatial strategies.

Parallel reaction monitoring (PRM) is a mass spectrometry technique used for the quantification and identification of targeted analytes in complex biological samples.^6,7^ PRM is valued for its high sensitivity, specificity, and accuracy in proteomic studies. For high-resolution PRM, precursor ions (at specific m/z and retention times) are isolated in a targeted way and fragmented. The resulting fragment ions were subsequently analyzed with high-resolution MS detectors, such as Orbitraps or time-of-flight detectors (Q-TOF)^7,8^. This allows for multiplexed simultaneous analysis of all fragment ions with high resolution and high accuracy (post-acquisition), in contrast to low-resolution single reaction monitoring (SRM) or multiple reaction monitoring (MRM) which typically require extensive optimization of Q1/Q3 transitions^9,10^. Due to the high resolution and post-acquisition refinement of fragment ion selection for quantification, PRM-MS generally is not as prone to interferences, and it is particularly efficient for fast developments of multiplexed assays. The innovative, spatial imaging parallel reaction monitoring (iprm) for MALDI-MSI, presented in this study, uses trapped ion mobility spectrometry (TIMS) to separate precursor ions based on their collisional cross section (CCS) values^11,12^. The addition of ion mobility separation to MALDI-MSI provides an orthogonal dimension of analyte separation, reduction of complexity and specificity to MSI experiments. The TIMS dimension allows for another level of isolation of precursor ions (analytes), specifically differentiating isomeric lipids/metabolites (with identical m/z but different CCS), in addition to the isolation based on the analyte m/z only prior to the spatial tandem mass spectrometry (MS/MS) imaging.

NAD^+^ (nicotinamide adenine dinucleotide) metabolism is of key relevance for maintaining cellular health, playing crucial roles in energy metabolism, DNA repair, transcriptional regulation, immune function and inflammation.^13,14^ Many clinical conditions implicate dysregulated NAD^+^ metabolism, including neurodegeneration, cancer, immune disorders, and cardio and metabolic diseases.^15–17^ Aging and age-related diseases and cellular conditions, such as cellular senescence, are also associated with declines in NAD^+^ and disruptions in NAD^+^-related metabolic processes, positioning NAD^+^ as a significant target for biomedical research.^17,18^ In the liver, reduced NAD^+^ levels have been associated with non-alcoholic fatty liver disease (NAFLD) and other age-related metabolic dysfunctions.^19^ CD38, is a potent enzymatic consumer of NAD^+^, and shows increased expression with age in several tissues.^13,18,20–22^ In liver tissue, CD38 is predominantly expressed in Kupffer cells, the resident macrophages of the liver sinusoids, where its activity drives cellular senescence and inflammation with aging.^23^ Thus, CD38 is considered as a major driver of the age-related NAD^+^ decline. Notably, CD38 knockout mice (CD38^−/−^) exhibit higher mitochondrial NAD^+^ levels in the liver and resistance to age-related NAD^+^ decline, suggesting improved metabolic regulation and protection against aging-associated metabolic deterioration.^20^

Additionally, NAD^+^ supplementation has emerged as a promising therapeutic strategy to enhance cellular function and mitigate age-related decline.^24,25^ Given the importance of hepatic NAD^+^ metabolism in systemic metabolic regulation, understanding how NAD+ and its related metabolites are spatially distributed within the liver could have significant therapeutic implications. We utilized our optimized iprm-PASEF assays to spatially map and precisely quantify lipids, NAD+, and related metabolites in liver tissues from wild-type (WT) and CD38 knockout (CD38^−^/^−^) mice. Here, we present our advanced multiplexed iprm-PASEF methodology, showcasing its capability for robust spatial MS imaging, identification, and accurate quantification of lipids and metabolites directly within liver tissue sections.

## Results

**Figure 1:**
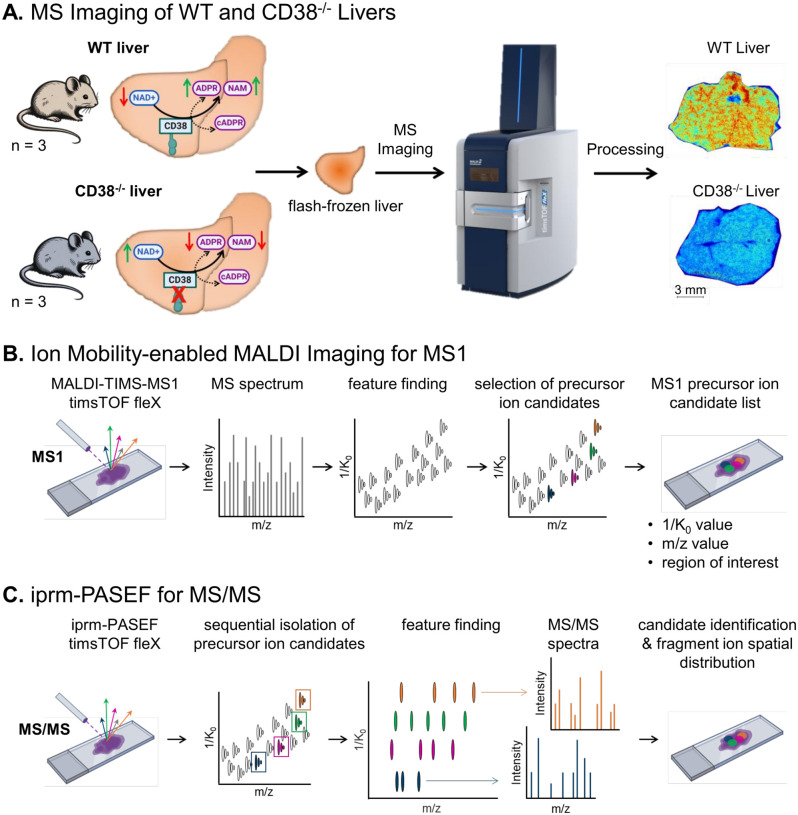
Experimental design for targeted MALDI-mass spectrometry imaging by iprm-PASEF of selected lipids and metabolites upon CD38 deletion in mouse liver. A. CD38 is a potent enzymatic consumer of NAD^+^ and CD38 knockout mice (CD38^−/−^) showed improved metabolism and lower cellular senescence in the liver, possibly through boosting the beneficial metabolic effects of NAD^+^. B. As a first discovery step, liver tissues from wild-type (WT) and CD38^−/−^ mice (n = 3 each) were subjected to collision cross section-enabled matrix-assisted laser desorption/ionization (MALDI)-mass spectrometry Imaging to spatially profile the lipidome and metabolome. Tissues sections were analyzed on a timsTOF flex, MALDI-2 mass spectrometer by MALDI-trapped ion mobility spectrometry (TIMS)-MS in both negative and positive ionization modes. Survey imaging scans were processed in Bruker SCiLS Lab using the T-ReX^3^ feature finding algorithm and the MetaboScape software. A list of precursor ion candidates, including lipids and metabolites, was generated. C. As a second validation step, targeted iprm-PASEF assays were developed and optimized in SCiLS Lab and timsControl software, extracting information on 1/K_0_, m/z and region of interest from the MALDI-TIMS-MS1 scans. Each precursor ion was ion mobility-aided isolated and *in situ* fragmented sequentially. Collected iprm-PASEF data were analyzed in SCiLS Lab to apply the feature finding algorithm and generate MS/MS scans. This enabled to confirm lipid and metabolite candidate identification, to map the spatial distribution of their fragment ions in the tissues, and to perform multiplexed MS2-based quantification of the fragment ions.

Fresh livers from three male, 18-month-old CD38^−/−^ and control (WT) mice were collected, flash frozen, sectioned onto glass slides and prepared for MS imaging with the Bruker timsTOF fleX MALDI-2 mass spectrometer. CD38^−/−^ animals were chosen for the spatial profiling of lipids and metabolites (Fig. 1A), due to their well-characterized increase in hepatic NAD^+^ levels with age compared to control (WT) counterparts^20^. Samples were first subjected to unbiased MS1 imaging in both negative and positive ionization modes for survey scans of the whole tissue cross-section. Preliminary identification of interesting candidate MS1 precursor ions, based on accurate m/z, isotope pattern, and ion mobility values (1/K_0_), was completed with the T-ReX^3^ feature finding algorithm in SCiLS Lab, and features were annotated directly in SCiLS by connecting to the MetaboScape 2025 REST API feature (Fig. 1B). To prioritize, candidate precursor ions that showed a predictive score difference of > 0.6 comparing CD38^−/−^ and WT liver tissues with the area under the receiving operator curve (AUROC) analysis were considered for the subsequent iprm-PASEF analysis. The iprm-PASEF target lists were assembled so that the candidate ions did not overlap in its 1/K_0_ range based on the MS1 survey scans. For the iprm-PASEF acquisitions each candidate precursor ion was isolated first by their ion mobility 1/K_0_ value in the TIMS cell, then by its m/z value in quadrupole Q1, and then finally fragmented in the Q2 collision cell. The MS/MS spectra were acquired in this targeted, sequential, and spatially resolved manner (Fig. 1C). All fragment ions for each analyte shared the same mobility isolation window as their precursor ion but had unique m/z values corresponding to lipid or metabolite fragment ions (as annotated in existing databases/spectral libraries, see methods). Spatial maps were generated for each fragment ion that matched the spatial distribution of the corresponding precursor ion.

**Figure 2:**
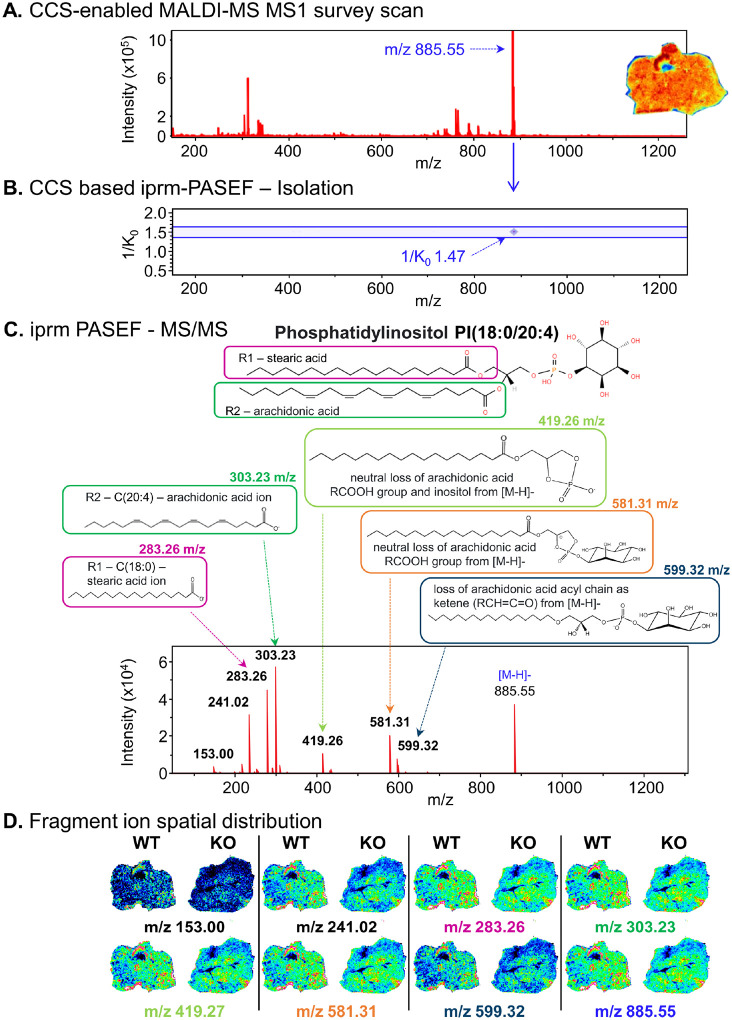
The iprm-PASEF strategy enables confident molecular identification with spatially resolved fragment ion distribution: example of Phosphatidylinositol PI(18:0/20:4). A collision cross section (CCS)-enabled survey MALDI-MS Imaging scan was acquired at the MS1 level to identify precursor ion candidates for targeted iprm-PASEF MS/MS-level analysis. Individual features of interest were selected based on their 1/K_0_ and m/z values. Up to 25 individual precursor ions, which were non-overlapping in 1/K_0_, could be simultaneously targeted for iprm-PASEF isolation and analysis. The iprm-PASEF MS/MS spectra for isolated and fragmented precursor ions were generated in a spatial manner in real-time. A. In CD38^−/−^ mouse liver, the composite survey MALDI-MS scan was acquired in negative ionization mode and showed the detection of a feature at m/z 885.55, that was highly abundant across the entire tissue section. B. This precursor ion candidate at m/z 885.55 and 1/K_0_ 1.47 VS/cm^2^ was selected for targeted iprm-PASEF isolation and fragmentation. C. The generated MS/MS spectrum showed species-specific fragment ions used for both accurate molecular identification and quantification of Phosphatidylinositol PI(18:0/20:4). The identified fragment ions were spatially mapped for representative images of wild-type (WT) and CD38^−/−^ mouse liver tissue sections based on their m/z. The spatial distribution of the PI(18:0/20:4) fragment ions matched the distribution of the corresponding precursor ion.

To demonstrate the ability for isolation and accurate identification of individual lipids and metabolites from complex biological matrices by iprm-PASEF, in our data sets, we first targeted a well-characterized and annotated feature at m/z 885.55 corresponding to phosphatidylinositol PI(18:0/20:4) as reported in previous studies.^26^ In negative ion mode this feature at m/z 885.55 was abundant in the MS1 spectra for all liver sections imaged (Fig. 2A). The collisional cross sections (CCS) that was observed for the m/z 885.55 in the MS1 survey scan showed a prominent feature at 1/K_0_ 1.47 Vs/cm^2^ (Fig. 2B). This feature was then isolated (**Supp.** Figure 1) based on its unique CCS (ion mobility) and m/z value combination allowing distinct analysis of this molecular feature separated from other features present in the liver tissue. The isolated candidate precursor ion was fragmented resulting in multiple abundant fragment ions. Importantly, the fragment ions all shared the same 1/K_0_ value as the isolated precursor ion, which allowed for generating a MS/MS spectrum of the fragmented [M-H]^−^ precursor ion accordingly (Fig. 2C). Fragment ions were annotated based on m/z values from existing databases and spectral libraries for lipid fragmentation. Major characteristic fragment ions corresponded to structures, such as stearic acid C(18:0) at m/z 283.26 and arachidonic acid C(20:4) at m/z 303.23 as well as several ions containing unique combinations of the phosphatidylinositol head group with the characteristic lipid tails at m/z 419.26, 581.31, and 599.32, among others (Fig. 2C). Combined, these confidently confirmed the identity of the precursor ion as PI(18:0/20:4). At the given MS/MS settings the precursor [M-H]^−^ ion at m/z 885.55 was still detectable even though at a significantly lower intensity than in the MS1 spectrum. MS/MS fragmentation was completed in a spatial raster manner at distinct tissue locations to generate the MS/MS imaging data set of fragment ions spatially across the liver sections (Fig. 2D). This showed that each fragment ion generated from one precursor ion displayed the same spatial distribution, sharing similar areas of high and low abundance across the tissue. Going forward, iprm-PASEF quantification can thus be performed on the fragment ion level for higher specificity and accuracy.

**Figure 3:**
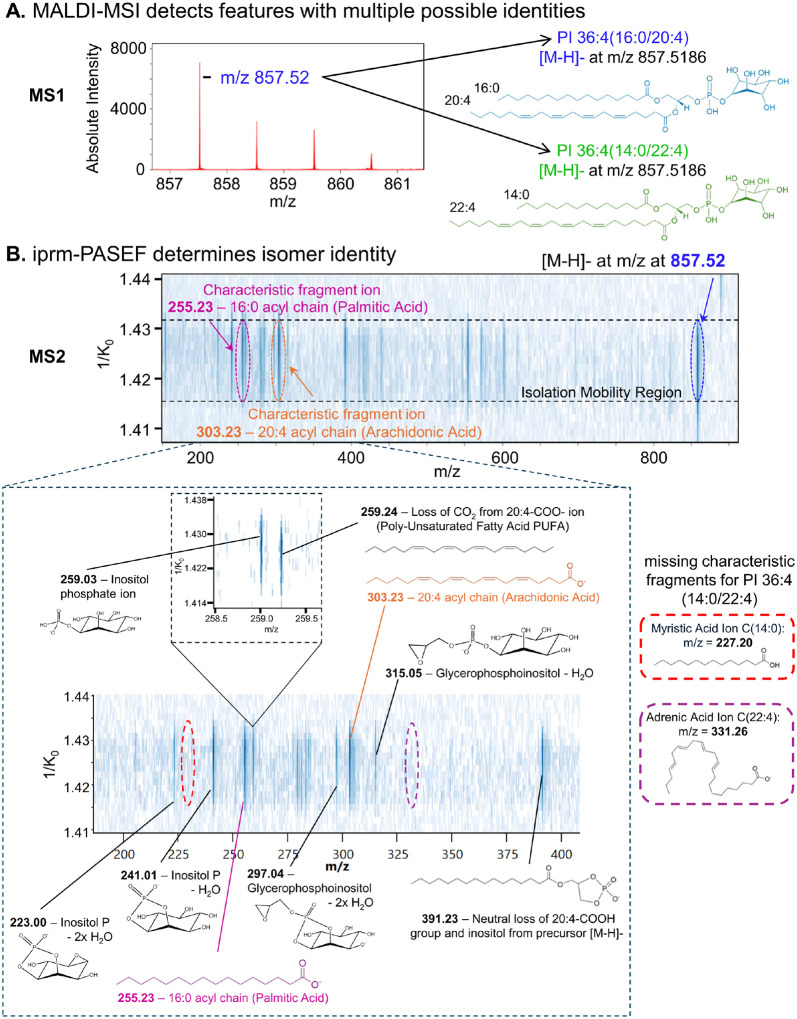
Detection of characteristic fragment ions for the spatially resolved identification of lipid isomers and isotopes in mouse liver tissues using iprm-PASEF. A. The zoom into the m/z range 857–861 of the composite survey MALDI-MS scan collected for CD38^−/−^ mouse liver tissue displayed an isotopic envelope for analytes (left). Potential lipid isomer discovery for the most intense ion at m/z 857.52 was initially annotated as PI (14:0/22:4) by MetaboScape software. B. The iprm-PASEF fragmentation pattern of the precursor ion [M-H]^−^ at m/z 857.52 in the mobility isolation window 1.4308 ± 0.0093 Vs/cm^2^ showing the detection of two characteristic fragment ions, m/z 255.23 (pink) and m/z 303.23 (orange), as evidenced for the identification of PI 36:4 (16:0/20:4). No presence of characteristic fragment ions for PI 36:4 (14:0/22:4) confirmed the absence of this species.

The survey MS1 scans revealed the regions in the composite MALDI-MS spectrum where different lipid isotope patterns were observed. One area of interest was the m/z range from m/z 857 to m/z 861 (Fig. 3A). Initial annotation (based on databases) of the intense peak at m/z 857.52 returned two possible isomeric phosphatidylinositols PI(36:4), each with 36 carbon atoms and a total of 4 unsaturated double bonds, PI 36:4(16:0/20:4) and PI 36:4(14:0/22:4). However, an overlapping isotopic distribution was observed, as the third isotopic peak (m/z 859.53) showed higher intensity than one would expect for a PI 36:4 (PI 36:4), suggesting the presence of a PI 36:3 (at m/z 859.53). Moreover, the spatial pattern for this ion at m/z 859.53, as determined by MALDI-MSI analysis, differed from that at m/z 857.52 (data not shown). The iprm-PASEF analysis was completed to ascertain the identities of these related lipid species. First, the feature at m/z 857.52 was isolated and fragmented for analysis. Characteristic fragment ions for PI 36:4(16:0/20:4) were detected in the isolated mobility window at 1/K_0_ 1.4308 ± 0.0093 Vs/cm^2^ corresponding to palmitic acid C(16:0) at m/z 255.23 and arachidonic acid C(20:4) at m/z 303.23 (Fig. 3B). Many other fragment ions were also detected that further confirmed the identity of this lipid, including several head group fragment ions validating the identity of the phosphatidylinositol lipid family (**Supp.** Figure 2). Some fragment ions detected in the iprm mobility window were unique to this MALDI-MSI iprm-PASEF experiment and did not match to annotated fragment ions from LC-MS analysis of this lipid. Also, characteristic fragment ions of the potential isomer, PI 36:4(14:0/22:4), including features at m/z 227.20 (myristic Acid 14:0) and m/z 331.26 (cholesterol ester 22:4), were not detected on the iprm mobilogram for this isolated feature (Fig. 3B). This enabled us to resolve its identity as PI 36:4(16:0/20:4) and confidently differentiate it from its potential isomer PI 36:4(14:0/22:4). Similar analysis was completed for the precursor ion at m/z 859.53 (**Supp.** Figure 3) identifying this feature as a combination of two different PI isoforms (for PI 36:3, 3 double bonds), specifically: PI (18:1/18:2) and PI (16:0/20:3), which were distinct from the two Dalton lower mass identified at m/z 857.52 as PI 36:4(16:0/20:4) with 4 double bonds as described in Fig. 3 above.

**Figure 4:**
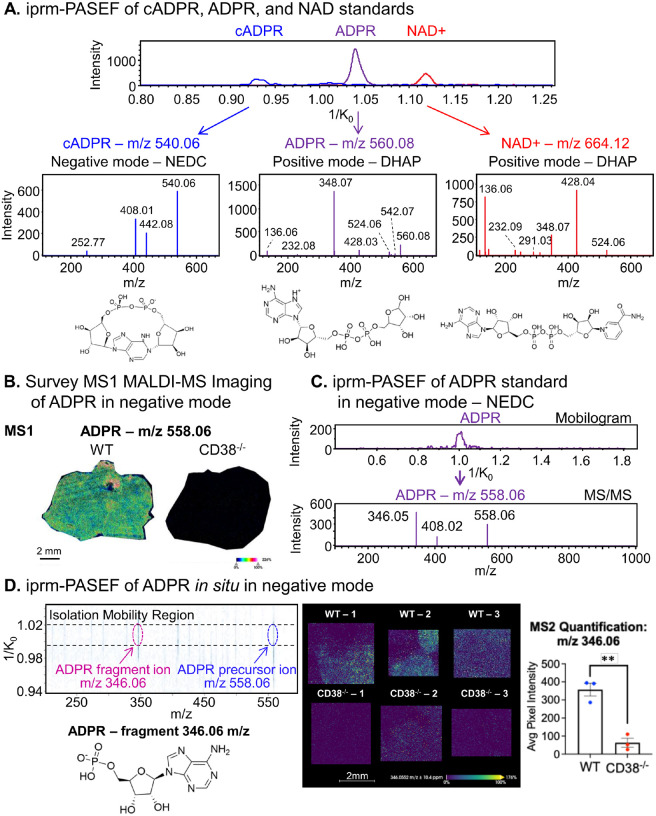
Confident identification of NAD+ and related metabolites in WT and CD38^−/−^ mouse livers by iprm-PASEF. A. The iprm-PASEF mobilogram and MS/MS spectra of synthetic cyclic adenosine 5′-diphosphate ribose (cADPR), ADPR, and nicotinamide adenine dinucleotide (NAD) standards. Each standard was individually analyzed: in negative ionization mode (N-(1-naphthyl) ethylenediamine dihydrochloride (NEDC) matrix) for cADPR (1 μM) with precursor ion [M-H]^−^ at m/z 540.06, in positive ionization mode (2,5-dihydroxyacetophenone (DHAP) matrix) for ADPR (10 μM) with precursor ion [M+H]^+^ at m/z 560.08 and for NAD^+^ (10 μM) with precursor ion [M+H]^+^ at m/z 664.12. B. Spatial distribution of ADPR precursor ion (negative ionization mode, NEDC matrix, [M-H]^−^ at m/z 558.06) for representative WT and CD38^−/−^ mouse liver sections analyzed by MALDI-MS Imaging. C. The iprm-PASEF mobilogram and MS/MS spectrum of the synthetic ADPR standard analyzed in negative ionization mode (1 μM) with precursor ion [M-H]^−^ at m/z 558.06. D. ADPR was selected for targeted iprm-PASEF analysis in predefined regions of interest of WT and CD38^−/−^ mouse liver tissue sections. The fragmentation pattern of ADPR precursor ion [M-H]^−^ at m/z 558.06 in the mobility isolation window 1.0045–1.0233 Vs/cm^2^ shows the detection of an ADPR fragment ion at m/z 346.06. This fragment ion was visualized *in situ* in the replicates of WT and CD38^−/−^ mouse liver and used for MS2-based quantification comparing average pixel intensity across the tissue sections. (ratio CD38^−/−^ vs WT = 0.18, p = 0.0025 meaning 5.6-fold downregulation in CD38^−/−^ vs WT, see **Supplemental Table 2**). ** Student’s t-test p-value < 0.01 (n = 3 individual mice/biological replicates per condition).

CD38 is a multifunctional enzyme with a predominant role as an NAD^+^ glycohydrolase, catalyzing the hydrolysis of NAD + to ADP-ribose (ADPR) and nicotinamide. Additionally, CD38 exhibits ADP-ribosyl cyclase activity, generating cyclic ADP-ribose (cADPR) from NAD^+^, though this constitutes a minor fraction of its total catalytic function. Furthermore, CD38 functions as a cADPR hydrolase, hydrolyzing cADPR to ADPR. Collectively, these enzymatic activities contribute to the regulation of NAD^+^ metabolism and calcium signaling, which are integral to cellular homeostasis. To identify optimal matrix conditions for MSI of NAD^+^ and its metabolites, we analyzed synthetic standards of NAD+, ADPR, and cADPR, individually spotted onto glass slides using various commonly used matrices. Dihydroxyacetophenone (DHAP) was used for positive ionization mode acquisitions and N-naphthylethylenediamine dihydrochloride (NEDC) was used for negative ionization mode. A representative mobilogram overview of the cADPR, ADPR, and NAD^+^ standards highlighted their distinct ion mobility drift times (1/K_0_ values) and indicated efficient separation in the TIMS dimension, while *in situ* fragmentation of the standards generated MS/MS spectra for each metabolite using iprm-PASEF (Fig. 4A). The fragmentation patterns obtained through iprm-PASEF showed strong concordance with those derived from conventional liquid chromatography-tandem mass spectrometry (LC-MS/MS) (**Supp.** Figure 4), where both methodologies detected characteristic fragment ions for each metabolite investigated.

Given that CD38 catalyzes NAD^+^ hydrolysis, we hypothesized that CD38^−/−^ liver would exhibit elevated NAD^+^ levels and a concurrent decrease in enzymatic by-products, such as ADPR and nicotinamide. To evaluate this, spatial MALDI MSI was performed for WT and CD38^−/−^ liver sections in negative ionization mode using NEDC matrix to monitor endogenous metabolites (n = 3 biological replicates per genotype). The MS1 survey scans revealed a marked reduction in ADPR (m/z 558.06) in CD38^−/−^ liver, with almost negligible signal intensity in CD38^−/−^ liver compared to WT liver (Fig. 4B). To further validate this observation, an ADPR standard was analyzed again, now in negative ionization mode (Fig. 4C) which confirmed its characteristic fragmentation pattern. The optimized endogenous iprm-PASEF assay, incorporating MS2 quantification of the characteristic ADPR fragment ion at m/z 346.06 *in situ*, demonstrated a significant spatial reduction in ADPR abundance in the CD38^−/−^ tissue samples compared to WT: with 5.6-fold downregulation in CD38^−/−^ across 3 biologically distinct replicates (Fig. 4D). These findings provided a proof-of-principle demonstration for CD38-mediated NAD^+^ metabolism in hepatic tissue and validated the iprm-PASEF approach as a robust technique for *in situ* spatial quantification of NAD^+^ metabolites at the MS2 fragment ion level.

**Figure 5:**
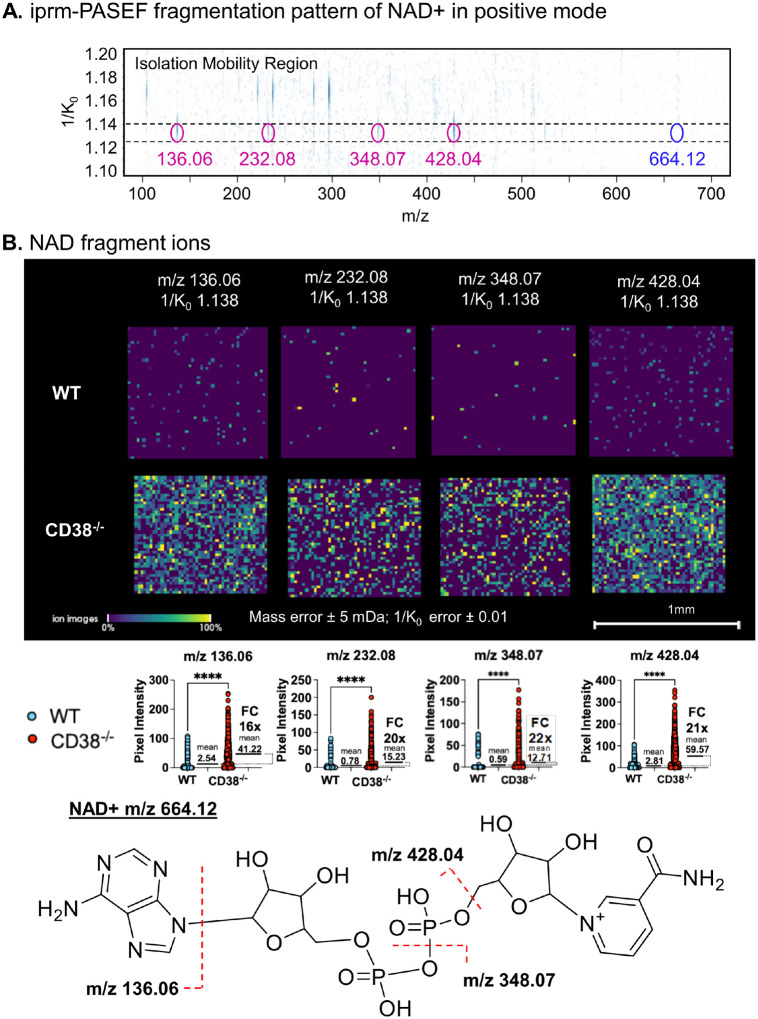
Quantification and *in situ* visualization of endogenous NAD+ fragment ions in WT and CD38^−/−^ mouse livers by iprm-PASEF. A. The iprm-PASEF fragmentation pattern of the endogenous NAD+ precursor ion [M+H]^+^ at m/z 664.12 in the mobility isolation window 1.1224–1.1400 Vs/cm^2^ showed the detection of fragment ions at m/z 136.06, m/z 232.08, m/z 348.07, and m/z 428.04. B. Spatial distribution of the four NAD^+^ fragment ions detected by iprm-PASEF in one WT and one CD38^−/−^ mouse liver replicate. Each fragment ion was quantified *in situ* using the pixel intensity within each tissue section, which all demonstrated an up-regulated level in CD38^−/−^ vs WT mouse livers. The statistical analysis was performed by comparing the MS2 intensity of each fragment ion in each pixel which composed the CD38^−/−^ vs WT image. **** Student’s t-test p-value < 0.0001 (n = 1885 pixels for the WT image and n = 2300 pixels for the WT image, see **Supplemental Table 3**; spatial tissue mapping for one mouse and one image replicate per condition). Fragmentation and cleavage sites of identified NAD^+^ fragment ions are mapped in the NAD^+^ structure.

Due to its highly polar and charged nature, NAD^+^ presents challenges with consistent ionization, which can impact detection sensitivity and quantification accuracy, particularly in the presence of ion suppression from lipids. In addition, NAD^+^ has in the past been shown to be prone to degradation thus this spatial imaging workflow may be advantageous due to very minimal sample handling^27^. In this study, we demonstrated efficient *in situ* detection and fragmentation of NAD^+^ in tissue using the iprm-PASEF approach. The iprm-PASEF workflow exhibited the capability to isolate and fragment the NAD^+^ precursor ion with [M + H]^+^ at m/z 664.12 in positive ionization mode with 2,5-dihydroxybenzoic acid (DHB) matrix (Fig. 5A). The presence of characteristic NAD^+^ fragment ions in the ion mobility isolation window, for the isolated precursor at m/z 664.12, including fragment ions at m/z 136.06, 232.08, 348.07, and 428.04 confirmed the identity of the isolated metabolite as NAD^+^ and subsequently allowed for accurate quantification. For the spatial imaging using iprm-PASEF, specifically, the MS2-level quantification and spatial imaging of each fragment ion revealed a significant increase in NAD^+^ levels in CD38^−/−^ liver tissue compared to WT, ~ 20-fold with very high statistical significance p < 0.0001 (Fig. 5B). These analyses further validated the role of CD38 in NAD^+^ metabolism and the robustness of the iprmPASEF technique for spatially resolved metabolite quantification.

## Discussion

The past decades have been marked by tremendous developments in omics technologies that have been instrumental for the in-depth exploration of complex biological systems, giving insights into molecular mechanisms and proposing novel clinical/therapeutic strategies. While very powerful, these technologies typically do not provide information about the native spatial context of biomolecules, which is highly relevant and critical to better appreciate the heterogeneity, architecture, and three-dimensional molecular organization of tissues. These needs have led to the rise of spatial omics technologies. Over the past five years, two spatial omics fields have been recognized as ‘Method of the Year’ by the journal *Nature Methods*, spatial transcriptomics in 2020^28^ and spatial proteomics in 2024^29^, demonstrating the powerful and crucial roles of these cutting-edge technologies to advance our understanding of biological complexity. Currently, even further advances are being made to improve the capabilities of these mass spectrometry imaging (MSI) techniques, especially in their ability to accurately identify and quantify complex biomolecules in place on tissue.

With respect to metabolomics and lipidomics, significant advances have been made in spatial mass spectrometry to investigate the spatial distribution of these molecules within tissues/organs. However, *in situ* identification remains very challenging. Here, we present the first study demonstrating the capabilities of imaging parallel reaction monitoring – Parallel Accumulation–Serial Fragmentation (iprm-PASEF) on lipids and metabolites for high-resolution, spatially resolved targeted MALDI-MS/MS Imaging. MALDI-MSI with iprm-PASEF technology allows for high accuracy analyte identification and quantification in real-time on the MS2 fragment ion level during imaging experiments. This novel workflow enables confident and accurate identification of complex and challenging molecules thanks to the acquisition of collision cross section (CCS)-enabled MS/MS spectra, while preserving their spatial information. Indeed, this strategy leverages the trapped ion mobility spectrometry (TIMS) technology to isolate molecular targets, first in the TIMS device based on their inverse reduced mobility (1/K_0_) and then in the quadrupole based on their mass-to-charge ratio (m/z). Isolated precursor ions are subsequently fragmented to collect high-resolution, full MS/MS spectra, while providing spatial information of both precursor and fragment ions. Ion mobility separation for lipid precursor ions is essential for proper identification and differentiation among many potential isoforms. MS2-level analysis further increases identity confidence through isolation and fragmentation of precursor ions of interest and subsequent detection of fragment ions that can be individually verified. The iprm-PASEF workflow utilizes the full capabilities of the timsTOF fleX and leverages the combination of ion mobility separation and precursor ion isolation and fragmentation to identify and quantify complex molecular features accurately. This workflow achieves label-free multiplexed and spatial multi-omic MS/MS imaging and quantification within a single acquisition.

The generated iprm-PASEF MS/MS spectra contain characteristic fragment ions that provide direct evidence for the *in situ* identification of lipids and metabolites present in tissues. The similar spatial distribution of the different fragment ions resulting from the same precursor ion provides an additional validation of the molecular identification. We showed that we can leverage the detection of characteristic fragment ions to decipher very complex and convoluted lipid profiles, as demonstrated for the well-studied and abundant Phosphatidylinositol PI(18:0/20:4) but also for more challenging and lower abundance features. The similarity in spatial patterns for identified fragment ions of PI(18:0/20:4) increased the confidence in isolated feature identification. Indeed, all fragment ions shared the same spatial pattern as the isolated precursor ion, signifying that all fragment ions were generated from a single precursor ion. Further, the ion mobility-resolved fragmentation pattern of the lipid PI 36:4(16:0/20:4) enabled highly-confident identification of this feature and enabled its differentiation from its structural isomer PI 36:4(14:0/22:4). This exemplified the ability for iprm-PASEF to accurately resolve lipids utilizing complex fragmentation patterns for multiple features across a dynamic abundance range. The ability to accurately identify analytes in place during iprm-PASEF experiments removes the need for orthogonal mass spectrometry technologies to confirm analyte identifications, significantly increasing the ability of MALDI-MSI in discovery-based studies.

Besides confident identification, we demonstrated that the iprm-PASEF strategy can achieve quantification at the MS2 fragment ion level, using spatially resolved fragment ions, enabling more specific and sensitive performances. Similar to targeted LC-MS PRM assays developed for targeted (non-spatial) proteomic approaches, iprm-PASEF experiments can be refined post-acquisition to select the most intense, non-interfered, specific and selective, fragment ions for the analytes of interest for their accurate and robust quantification, while additionally providing a spatially defined context. This represents an asset in the context of complex and convoluted metabolome and lipidome analysis, as fragment ions differentiating co-fragmented species can be specifically used to ensure accurate quantification. We developed and optimized novel iprm-PASEF assays for *in situ* quantification of endogenous NAD and ADPR metabolites in mouse livers for. Through confident identification of these highly selective fragment ions, we demonstrated the ability for high confidence, specificity, and accuracy quantification for lipids and metabolites during MALDI-MSI experiments on the MS2 fragment ion level.

Our application of the iprm-PASEF workflow to CD38 and NAD^+^ biology demonstrates its power to better understand the underlying molecular mechanisms and address biological questions. NAD^+^ is a central redox coenzyme critical for cellular metabolism, supporting key oxidative and reductive reactions and serving as a substrate for NAD^+^-consuming enzymes such as sirtuins, PARPs, and the NADase CD38. With age, NAD^+^ levels decline in multiple tissues, including the liver and ovary, contributing to mitochondrial dysfunction and impaired metabolism^13,18,20–22^. Our findings, along with independent studies, implicate CD38 as a major driver of this decline through its NADase activity, which hydrolyzes NAD^+^ primarily into nicotinamide and ADP-ribose (ADPR)^13,18,20–22^.

While global (bulk) NAD^+^ quantification by LC-MS is well established, understanding its spatial distribution is essential for elucidating compartment-specific metabolic regulation of NAD^+^ and related metabolites. Mass spectrometry imaging offers spatial resolution but - in some cases - faces challenges in detecting small, labile metabolites like NAD^+^ and ADPR due to ion suppression from abundant proteins and lipids, as well as difficulties in metabolite annotation. To overcome these limitations, prior studies have employed HRMS/MS and spiking of NAD^+^ standards onto control tissue^30,31^. For instance, Lu *et al*. demonstrated that acidified methanol washes reduce ion suppression and, when combined with isotopic labeling, allow confident identification of NAD^+^ and related metabolites^32^. In this study, we utilized the iprm-PASEF strategy to map the spatial distribution of NAD^+^ and ADPR in wild-type and CD38 knockout mouse livers. Using refined fragment ions”, *i.e*. the fragment ion at m/z 346.06 for ADPR (in negative ionization mode) and the panel of fragment ions at m/z 136.06, m/z 232.08, m/z 348.07, and m/z 428.04 for NAD^+^ (in positive ionization mode), we were able to obtain spatially resolved MS2-based quantification and further conduct statistical analysis to confirm their highly significant increase upon CD38 deletion in mouse livers. Additionally, we optimized matrix formulations utilizing both DHAP and DHB to enhance NAD^+^ ionization during MALDI MS Imaging experiments from both slide-spotted standards and directly from tissue sections, further improving MSI sensitivity and reliability.

We envision complementary, synergistic, and multi-modal approaches combining global LC-ESI-MS/MS and spatial MALDI-MS/MS (iprm-PASEF) Imaging to i) generate ESI-based spectral libraries to help interpret MALDI-MS/MS spectra and improve identification rate in MS-imaging assays, ii) inform on the spatial distribution of analytes of interest identified by ESI-MS/MS experiments, iii) aid in integrating multi-omics measurements and data. This novel iprm-PASEF workflow opens the way to novel strategies for advanced spatial metabolomics, lipidomics, and proteomics – including *in situ* tryptic peptides^33^ or any other proteolytic peptides/proteins, that will be pivotal in obtaining a more holistic and refined picture of the dynamic organization and regulation of biological systems. With the multiplexed capabilities of iprm-PASEF in performing high-quality identification and simultaneous quantification for multiple complex lipids and biologically relevant small molecule metabolites, we have demonstrated the importance and future opportunities of MS2-based spatial mass spectrometry imaging. Specific future interests could be in spatial mapping of tissues highlighting therapeutic drug distribution, regional biomarker responses upon interventions, and overall tissue heterogeneity during aging and age-related diseases.

## Methods

### Chemicals and Samples

Formic acid, acetonitrile (ACN), trifluoroacetic acid (TFA), methanol, water (all LCMS grade), 2,5-dihydroxyacetophenone (DHAP), 2,5-dihydroxybenzoic acid (DHB) N-naphthylethylenediamine dihydrochloride (NEDC), and chemical standards for nicotinamide adenine dinucleotide (NAD+), ADP-ribose (ADPR) and cyclic ADP-ribose (cADPR) were purchased from Sigma-Aldrich (St. Louis, MO, USA). Livers from mice with global deletion of CD38 (CD38^−/−^) and control, litter mate wild-type (WT) mice (18 months old) were collected at the Buck Institute for Research on Aging.^20^

### Sample preparation for spatial iprm-PASEF imaging

Livers were collected from CD38^−/−^ mice and WT controls (n = 3 biological replicates each) and flash frozen with liquid nitrogen to preserve lipids and metabolites. Frozen livers were mounted directly to a cryotome (Leica, Germany) pre-chilled to −20°C using a cellulose-based mounting media (M-1 Embedding Matrix, Epredia, Kalamazoo, MI). The portal vein was used to standardize the orientation of the left major lobe of each liver. Tissue sections with 10–12 μm thicknesses were mounted on charged microscope slides. Tissues slides were desiccated and then stored under vacuum at − 80°C prior to matrix application. Chemical standards for NAD^+^, ADPR, cADPR were dissolved in methanol at a concentration of 10 μM or 1 μM and spotted onto bare microscope slides with a pipette. Slides were desiccated to evaporate methanol. The HTX TM Sprayer M3 was used to apply matrix by aerosol^34^. For positive ionization mode experiments, 15 mg/mL DHAP or DHB in 80% ACN, 10% methanol in water was applied with the following parameters: flow rate 120 μL/min, number of passes 6, temperature 70°C, nitrogen gas pressure 10 psi, pattern CC, velocity 1200 mm/min, drying time 5 s. For negative ionization mode experiments, 7 mg/mL NEDC in 70% methanol in water was applied with the following parameters: flow rate 150 μL/min, number of passes 10, temperature 70°C, nitrogen gas pressure 10 psi, pattern HH, velocity 700 mm/min, drying time 0 s.

### MALDI-MS Imaging Analysis

#### MALDI-TIMS-MS1 acquisitions

MALDI-MS Imaging was performed using a Trapped Ion-Mobility Time-of-Flight Mass Spectrometer, the timsTOF fleX MALDI-2 (Bruker Scientific, LLC, Bremen, Germany) equipped with a dual ESI/MALDI source. Survey MS1 scans were acquired with single-scan spectra consisted of 200 accumulated laser shots at 1 kHz. MALDI-MS images were acquired at a 20 μm pixel resolution for both polarity ion imaging experiments. The SmartBeam 3D 10 kHz laser was set to 35% power, scan range of 16 μm for X and Y and resulting field size of 20 μm for X and Y. Additional instrument parameters for the studies included: an ion transfer time of 50 μs, a pre pulse storage time of 60–80 μs, a collision RF of 800–1000 Vpp, a collision energy of 10 eV, an ion energy in the quadrupole of 5.0 eV, a TIMS funnels 1 RF and 2 RF of 250 Vpp, and a multipole RF of 250 Vpp. Negative ionization mode acquisitions targeting lipids ranged from m/z 150–1100 and 1/K_0_ 0.4–1.8 VS/cm^2^ with a 100-m/s ramp time. Positive ionization mode acquisitions, tuned for small molecule metabolites specifically, ranged from m/z 100–1000 and 1/K_0_ 0.4–1.4 VS/cm^2^ with a 200-ms ramp time.

#### iprm-PASEF acquisitions

iprm-PASEF was performed in both positive and negative ionization acquisition modes. Main instrument settings were not adjusted between MS1 and MS2 acquisitions. Both modes used a mobility-based isolation strategy that isolated precursor ions by a 1/K_0_ window first followed by isolation by m/z. Collision energy was determined by a linear fit based on precursor mobility ranging from 35 eV at 1/K_0_ 0.8 VS/cm^2^ to 60 eV at 1/K_0_ 1.6 VS/cm^2^. No isolated precursor ions had a mobility greater than 1/K_0_ 1.6 VS/cm^2^. iprm-PASEF precursor isolation settings including m/z and mobility ranges can be found in **Supplemental Table 1**.

#### MALDI-MSI data analysis

After MS acquisition, the data was imported into SCiLS Lab Pro v2025a (Bruker Scientific, LLC, Bremen, Germany) for initial MS1 data visualization and iprm-PASEF target list assembly. Metaboscapev.2025, (Bruker, Germany) was used to annotation analytes using existing or customized libraries to build precursor ion list based on m/z, isotopic pattern, and ion mobility values. Subsequently, iprm-PASEF data was used to confirm analyte identification by comparing fragmentation spectra to LipidMaps and the MSDIAL-TandemMassSpectralAtlas for both positively- and negatively-charged analytes. Data was analyzed using SCiLS Lab Pro v2025a and Data Analysis v4.0 software (Bruker, Germany). iprm-PASEF data was further used for MS2-level quantification of selected fragment ions. A statistical analysis comparing the pixel intensity of the fragment ions was performed in Prism v10.4.2 (GraphPad), using a Student’s t-test. Quantitative results used for statistical analysis can be found in **Supplemental Tables 2–3**.

### LC-MS/MS Analysis

Confirmation of NAD^+^ and ADPR was performed by direct LC-MS analysis of pure standards obtained from Sigma Aldrich (N0632 for NAD^+^ and A0752 for ADPR). Standards at 10-micromolar concentration were injected onto a Thermo Hypercarb column (5 μm, 100 × 2.1 mm) using a Vanquish UHPLC system. Chromatographic separation was carried out over a 12-minute gradient using solvent A (7.5 mM ammonium acetate with 0.05% ammonium hydroxide in water) and solvent B (0.05% ammonium hydroxide in acetonitrile) as follows: 0–1 min, 5% B; 1–6 min, linear gradient to 60% B; 6.1–7.5 min, 90% B; 7.6–12 min, re-equilibration at 5% B. Mass spectrometric detection was performed on a Thermo Q Exactive mass spectrometer operated in positive ion mode using a targeted SIM-PRM acquisition strategy. Targeted SIM (tSIM) scans were acquired across a mass range of m/z 50–750 at 70,000 resolution, AGC target of 1e6, and maximum injection time of 100 ms. PRM scans were performed at a resolution of 17,500, with an AGC target of 5e5, maximum injection time of 55 ms, and stepped normalized collision energies (NCE) of 20, 35, and 30. Data were visualized using Thermo Freestyle software.

## Supplementary Material

Supplementary Files

This is a list of supplementary files associated with this preprint. Click to download.

- AdditionalInformationlegends.docx
- SuppFigure1iprmisolation20250421v4.pptx
- SuppFigure2iprmlipid364MSMS0421v6.pptx
- SuppFigure3coisolation0421v5.pptx
- SuppFigure4LCMSMSADPRcADPR250421v6.pptx
- SupplementalTable1iprmIsolationSettingsv2.xlsx
- SupplementalTable2ADPRFragmentIonQuantificationv3.xlsx
- SupplementalTable3NADFragmentIonsQuantificationv3.xlsx

## Data Availability

The spatial proteomic and liquid chromatography mass spectrometry raw MS data sets have been uploaded to the Center for Computational Mass Spectrometry, MassIVE, and can be downloaded using the following FTP link: ftp://MSV000097855@massive-ftp.ucsd.edu or via the MassIVE website: https://massive.ucsd.edu/ProteoSAFe/private-dataset.jsp?task=4dbd4a7e3a4d4f66b90399a693974e2f(MassIVE ID: MSV000097855). [Note to the reviewers: To access the data repository MassIVE (UCSD) for MS data, please use: Username: MSV000097855_reviewer; Password: winter].
